# Complete Genome Sequence of a *Klebsiella pneumoniae* Isolate with Chromosomally Encoded Carbapenem Resistance and Colibactin Synthesis Loci

**DOI:** 10.1128/genomeA.01332-14

**Published:** 2014-12-24

**Authors:** Sean Conlan, Clayton Deming, Yu-Chih Tsai, Anna F. Lau, John P. Dekker, Jonas Korlach, Julia A. Segre

**Affiliations:** a National Human Genome Research Institute, Bethesda, Maryland, USA; b Pacific Biosciences, Menlo Park, California, USA; c National Institutes of Health Clinical Center, Bethesda, Maryland, USA

## Abstract

*Klebsiella pneumoniae* is an important nosocomial pathogen, and multidrug-resistant strains have become a worldwide concern. Here, we report the complete genome of a *K. pneumoniae* isolate with chromosomally integrated *bla*_KPC_ genes and a colibactin synthesis locus.

## GENOME ANNOUNCEMENT

*Klebsiella pneumoniae* is a Gram-negative bacterium and a major cause of nosocomial infections. Of particular concern is the rise of carbapenem resistance in *K. pneumoniae* via the acquisition of plasmid-encoded β-lactamase *bla*_KPC_ (*K. pneumoniae* carbapenemase [KPC]) genes. Here, we report the complete genome sequence of a *K. pneumoniae* isolate carrying three copies of the *bla*_KPC_ gene, two of which are integrated into the bacterial chromosome.

*K. pneumoniae* strain KPNIH33 was isolated from a patient who was referred to the NIH Clinical Center for the treatment of metastatic colon cancer. Epidemiological evidence and preliminary shotgun sequencing data indicated that this patient had acquired carbapenem-resistant *K. pneumoniae* prior to his referral to our hospital. The KPNIH33 isolate grew from a urine culture collected in an outpatient clinic in August 2013, before the patient had ever been admitted to the NIH Clinical Center. The complete genome sequencing of KPNIH33 was undertaken as part of a larger study of plasmid diversity across carbapenem-resistant *Enterobacteriaceae* (1).

KPNIH33 genomic DNA was sheared to ~20 kb using Covaris g-tubes (Covaris) and converted into SMRTbell template libraries. The library was size selected with a lower cutoff of 4 kb using BluePippin. Sequencing was performed on the PacBio RSII using P4 polymerase binding and C2 sequencing kits with 180-min acquisition. The reads were assembled using HGAP (2) and Quiver (SMRTAnalysis version 2.0).

The KPNIH33 genome consists of a 5,574,202-bp chromosome and three plasmids (75,618 bp, 36,109 bp, and 9,294 bp). The chromosome has 5,710 predicted protein-coding sequences, 8 rRNA operons, and 88 tRNAs. KPNIH33 belongs to sequence type 258, the dominant sequence type of *K. pneumoniae* in hospitals. The 75.6-kb plasmid is closely related to the pBK30661 plasmid (3), differing by two indels and 46 high-quality single-nucleotide variants (SNVs). The 36.1-kb plasmid is nearly identical to plasmid pNJST258N3 (2 high-quality SNVs: C1413A and G34604A) from another sequence type 258 (ST258) *K. pneumoniae* isolate (4). The small 9.3-kb plasmid is identical to pEA1509_B from an *Enterobacter aerogenes* clinical isolate (5).

The KPC β-lactamase gene is typically found on plasmids (6) and is a significant source of carbapenem resistance within *Enterobacteriaceae* (7). KPNIH33 carries a KPC-3 allele on the 75.6-kb plasmid in the context of a truncated Tn*4401*/Tn*1331* nested transposon. In addition, KPNIH33 has two chromosomal *bla*_KPC-3_ genes arranged as an inverted repeat of two Tn*4401*d transposons separated by 433 bp. Chromosomally encoded *bla*_KPC_ is unusual; however, manual inspection of the assembly showed clear long-read sequence level support of the junctions between the Tn*4401*d cassettes and the chromosome. In addition, the integrated *bla*_KPC_ genes were confirmed using PCR designed to test the junctions between Tn*4401*d and the chromosome.

A second unusual feature of KPNIH33 is the insertion of 130 kb of sequence that is 99% identical to the *Citrobacter koseri* BAA-895 yersiniabactin (a siderophore-dependent iron uptake system) and colibactin *pks* loci. Colibactin is a genotoxic nonribosomal peptide that induces DNA double-stranded breaks in eukaryotic cells, resulting in cell cycle arrest (8). In the survey of isolates conducted by Putze et al. (9), the *pks* pathogenicity island was found in 3.5% of the *K. pneumoniae* isolates.

### Nucleotide sequence accession numbers.

The complete genome and plasmids for *K. pneumoniae* KPNIH33 are available in Genbank under accession numbers CP009771, CP009772, CP009773, and CP009774 (BioProject PRJNA243858).
